# Are dietary patterns in early childhood associated with alcohol consumption at the age of 17 years? Analysis of data from the Avon Longitudinal Study of Parents and Children (ALSPAC) prospective cohort study

**DOI:** 10.1017/S1368980021004183

**Published:** 2022-09

**Authors:** Katherine Yorke, Kate Northstone, Louise R Jones

**Affiliations:** Population Health Sciences, Bristol Medical School, University of Bristol, Oakfield House, Oakfield Grove, Bristol BS8 2BN, UK

**Keywords:** ALSPAC, Principal component analysis, Adolescence, Alcohol, Sugar

## Abstract

**Objective:**

To examine the relationship between a posteriori dietary patterns in early childhood and alcohol consumption in adolescence.

**Design:**

Data were obtained from the Avon Longitudinal Study of Parents and Children (ALSPAC) prospective cohort study. Dietary information was obtained using FFQ at the age of 3 and 7 years. The association between dietary patterns, derived using principal components analysis and the Alcohol Use Disorders Identification Test (AUDIT) scores (to assess harmful intake) and frequency of alcohol consumption at the age of 17 years were examined. Secondary analysis considered sugar intake as a percentage of total energy intake.

**Setting:**

Women who gave birth between 1 April 1991 and 31 December 1992 in the Avon area in southwest England were eligible for the ALSPAC cohort study.

**Participants:**

Totally, 14 541 pregnancies were enrolled in ALSPAC during its initial recruitment phase. For this analysis, complete data were available for between 3148 and 3520 participants.

**Results:**

Adherence to the ‘healthy’ dietary pattern at both 3 and 7 years of age was positively associated with consuming more than one alcoholic drink per week at 17 years of age, whilst adherence to the ‘traditional’ dietary pattern at both ages was protective of harmful alcohol intake at 17 years of age. Sugar intake was not associated with either alcohol outcome after adjustment for ethnicity, maternal level of education, parental social class and maternal AUDIT score.

**Conclusions:**

For the population studied, changes to diet in early childhood are unlikely to have an impact on harmful alcohol use in adolescence given the lack of consistency across the results.

Alcohol use is a leading cause of mortality and morbidity worldwide^(1–3)^. The 2016 Global Burden of Disease identified alcohol as the leading risk factor in male and female deaths aged 15–49 years in 2016, and the seventh leading risk factor for all ages for deaths and disability-adjusted life years^(1)^. Alcohol use also places a high burden on healthcare systems, costing the National Health Service (NHS) in the UK an estimated £3·5 billion per year^(4,5)^. Alcohol consumption most commonly begins in adolescence in countries with a high prevalence of alcohol use^(6,7)^. A recent study in the UK identified that 90 % of young people within their study population had their first taste of alcohol by the age of 17 years^(6)^. In addition, it has been shown that young people who binge drink in adolescence are more likely to report binge drinking as young adults^(8)^ and to be exposed to greater alcohol harm as they grow older^(7,9)^. Understanding the factors that contribute to higher alcohol consumption in the adolescent population is a key part of longer-term strategies for reducing population level alcohol harm in adults^(8)^. Identifying sensitive periods where early intervention may have an impact is important for the development of appropriate interventions both in childhood and in adolescence^(8,10)^. There is no single contributory factor to a young person’s propensity to over-consume alcohol^(7)^. However, each of the factors that do contribute merit attention as they could prove to be influential in identifying points for intervention.

There is evidence to suggest that sugar and alcohol addiction may be related^(11)^. Studies in animals have identified that higher consumption of sugar, and possibly fat, may mimic some properties of addictive substances^(12,13)^. In particular, binging of sugar and fat becomes more pronounced in rodents the longer that they are exposed to increased levels of sugar and fat (i.e. levels in excess of what would normally be fed in a laboratory environment) on a regular basis^(13)^. Animal studies have also shown that exposing rats to cycles of increased sugar in the diet followed by complete withdrawal of sugar causes a similar neurochemical response to opiate withdrawal^(12,14)^, suggesting that sugar may be addictive. Understanding how this research may apply to humans is complex. Firstly, if certain foodstuffs were found to be addictive, whether a human becomes addicted to them is likely to be multifactorial^(10,15)^. In addition, linking this to other addictive substances or behaviours, such as patterns of alcohol use or addiction, adds further complexity and identifying causality is very challenging. Some addictive behaviours have been studied alongside diet and, for example, it has been identified that people with high scores on the Yale–Brown Obsessive Compulsive Scale – modified for Pathological Gambling (YPG-YBOCS) – also had higher total fat intake than others included in the study^(16)^. Biological children of parents with alcohol dependence are more likely to over-consume sugar, which indicates an association in the opposite direction^(12)^.

Recent research has identified that dietary intake in childhood may be a contributing factor to alcohol use in adolescence. A 2018 study by Mehlig *et al.* ^(10)^ using data, the Identification and prevention of Dietary-and lifestyle-induced health Effects in Children and infantS (IDEFICS)^(10)^, showed that children with a high propensity to consume sugar and fat were at 2·46 times at greater risk (RR: 2·46; (95 % CI 1·47, 4·12)) of alcohol consumption in adolescence compared to those with low propensity, after adjustment for confounders^(10)^.

The literature on children’s dietary intake and later alcohol consumption is limited. To contribute to research in this area, we used data from the Avon Longitudinal Study of Parents and Children (ALSPAC), a prospective UK cohort study. Given the fact that nutrients are not consumed in isolation^(17)^, we wanted to examine the association between dietary patterns, looking at the diet as a whole in early childhood with later alcohol consumption. The aim of the study was therefore to examine the associations between dietary patterns in early childhood and alcohol intake and harmful behaviour at the age of 17 years. In addition to patterns, we specifically identified sugar as a factor of interest^(10)^. We hypothesised that children following a dietary pattern high in ‘processed’ food or sugar would be more likely to develop harmful alcohol behaviours in adolescence and more likely to consume alcohol at a higher frequency than those children adhering to a dietary pattern deemed ‘healthy’ or ‘traditional’ or having low sugar consumption.

## Methods

### Participants

Pregnant women resident in Avon, UK, with expected dates of delivery 1 April 1991 to 31 December 1992 were invited to take part in ALSPAC. The initial number of pregnancies enrolled was 14 541. Of these, there were 14 062 live births and 13 988 children alive at 1 year of age^(18,19)^. Data were primarily collected using self-completed questionnaires which were administered during pregnancy and at set ages once the child was born^(20,21)^. The ALSPAC participants included in this study were those who had dietary intake data available at both 38 months and 81 months, as well as alcohol use data at the age of 17 years. Ethical approval for the study was obtained from the ALSPAC Ethics and Law Committee and the Local Research Ethics Committees. Informed consent for the use of data collected via questionnaires and clinics was obtained from participants following the recommendations of the ALSPAC Ethics and Law Committee at the time. Please note that the ALSPAC study website contains details of all the data that is available through a fully searchable data dictionary and variable search tool: http://www.bristol.ac.uk/alspac/researchers/our-data/.

### Measurement of exposures

Dietary intake was measured using a FFQ when the children were aged 38 months and 81 months. The main carer was asked to provide information on how regularly their child consumed a wide range of items, including everyday basic foods as well as snacks and drinks (thirty-four items at 38 months and four-one items at 81 months). The FFQ gave the following options for response: (i) never or rarely; (ii) once in 2 weeks; (iii) 1–3 times a week; (iv) 4–7 times a week or (v) more than once a day^(20)^. Portion sizes were not asked about. For everyday items, such as milk and bread, more detailed questions were asked, for example, the number of slices of bread per d and the type of bread^(20,22)^. Dietary patterns were obtained using principal component analysis (PCA) at each time point^(20–22)^. In brief, frequency of consumption options were converted to times per week as follows: (i) 0; (ii) 0·5; (iii) 2; (iv) 5·5 and (v) 10 times per week. All items were standardised by subtracting the mean and dividing by the standard deviation for each variable. The data were then entered into a PCA with a Varimax rotation^(23,24)^, and the number of components that best represented the data were chosen using a scree plot. A score for each child was calculated for each component identified at each time point, each score has a mean of 0 and a standard deviation of 1 in the population in which it was derived and a higher score indicates a higher adherence to that dietary pattern.

Non-milk extrinsic sugar (NMES) (free sugars in the diet not including milk) intake was estimated from the FFQ as grams per week based on the fifth edition of McCance & Widdowson’s The Composition of Foods^(25)^. The estimated intake was adjusted to account for overall energy intake for each participant and is presented as the percentage of overall energy intake. We have previously shown that estimated sugar intake was strongly correlated with the ‘processed’ pattern at 3 (previously labelled ‘junk’) and 7 years of age^(22)^ (*r* = 0·475 and 0·637, respectively).

### Measurement of outcomes

There were two outcome measures used in the study. Alcohol consumption was measured at the age of 17 years using a computerised questionnaire, which was completed by the young person in a clinic setting. The questionnaire included the Alcohol Use Disorders Identification Test (AUDIT)^(26)^ and AUDIT score was used as the first outcome measure. Clinically, a score of 8 or more in the AUDIT test is considered to indicate an increased risk of harm from alcohol^(26,27)^, and we therefore chose to use this dichotomy.

The second outcome was a measure of the frequency of alcohol consumption and was based on responses to a question that asked how often the young person had a drink containing alcohol. The options were never, monthly or less, 2 or 4 times a month, and 2 or 3 times a week or 4 or more times a week. We collapsed the five categories into ‘at least weekly’ (‘2 or 3 times a week’ and ‘4 or more times a week)’ compared with less than weekly: (‘never’, ‘monthly or less’ and ‘2 or 4 times a month’) in order to facilitate comparison with the IDEFICS study^(10)^.

### Statistical methods

STATA v. 15.1 was used to perform logistic regression to assess the relationship with each of the exposure variables and both outcomes. OR and 95 % CI are presented. The exposure variables were categorised into quintiles due to our interest in the extremes of each dietary pattern. This also enhanced interpretation of any associations (i.e. making comparisons to the lowest quintile rather than a unit-less continuous variable).

A Direct Acyclic Graph (DAG) diagram was used to identify potential confounders from the available data. Relevant literature was consulted while developing the DAG to inform the decision on which variables could be potential confounders and should be included. For example, alcohol harm^(28)^ and poor nutrition^(29)^ are more prevalent in deprived communities and therefore social class was included as a potential confounding variable. Based on this approach, four confounding factors were included in the adjusted analysis which were the child’s ethnicity, mother’s highest educational qualification, household social class and the mother’s AUDIT score. There is evidence that there is a difference in the reporting of levels of alcohol consumption between girls and boys, and therefore an interaction test for gender was completed^(10,30)^.

Multiple imputation by chained equation was used to impute missing data with the use of the ice command in STATA^(31)^. Twenty-five datasets were generated and ten switching procedures were undertaken. The results can be seen in Supplemental Tables A–D. The following variables were used to impute all outcomes, main predictors, and other variables included in the adjusted analyses plus the Family Adversity Index score, which is comprised of early parenthood, housing (adequacy, basic living facilities (e.g. hot water) and defects), financial difficulties, partner (present/absent), relationship with partner (affection/cruelty/support), family size, major family problems (child in care, not with natural mother or on at risk register), maternal depression/anxiety, substance abuse and crime (trouble with police or convictions). The analyses of associations between dietary patterns and sugar intake with AUDIT and number of drinks consumed were then repeated with the imputed data.

## Results

PCA was completed on all participants with dietary data at each time point (*n* 10139^(20)^ and *n* 8286^(21)^ at 38 and 81 months, respectively). AUDIT score data were available for 4148 at the age of 17 years. Scores ranged from 0 to 40 with a median of 6 and a mean of 7 (sd 4·8). Alcohol consumption data were available for 3969 young people at the age of 17 years. Data on both dietary intake and alcohol consumption were available for between 3148 and 3520 of the original cohort in the unadjusted analysis.

PCA identified four major dietary components from the FFQ at 38 months, which accounted for 23·5 % of the variation within the sample^(20)^. Each child was given a score for the four components to indicate their predominant dietary pattern or patterns^(20)^. The four components identified were ‘processed’ (note that in previous papers this was referred to as ‘junk’, high positive loadings for snack foods and foods high in fat such as crisps, sweets, biscuits, chocolate sausages, burgers, chips and takeaway foods), ‘healthy’ (high positive loadings for vegetables, fruit, rice, pasta, pulses and meat substitutes), ‘traditional’ (high positive loadings for meats, poultry, potatoes and vegetables) and ‘snack’ (high positive loadings for finger foods such as bread, fruit, biscuits, crisps and cheese). Analysis at 81 months identified three dietary patterns, ‘processed’, ‘healthy’ and ‘traditional’, which had very similar loadings for foods as the 3-year patterns and explained 18·2 % of variation within the sample^(21)^. The data available on NMES intake was adjusted to account for overall energy intake for each participant and is presented as a percentage of total energy intake. The percentages for NMES ranged from 0·6 % to 39·8 % at 38 months, and 1·1 % to 40·3 % at 81 months.

Associations were evident between each of the confounders considered and at least one of the outcomes (see Table 1). An AUDIT score ≥ 8 was more likely in children of White ethnicity and those whose mothers also reported an AUDIT score ≥ 8. Adolescents who reported consuming more than one drink per week were more likely to have lower educated mothers, be from White backgrounds and have mothers with a high AUDIT score.

**Table 1:** Association between dietary pattern scores and confounders with outcome measures; *n* (%) for categorial variables, mean (sd) for continuous variables

	AUDIT score					Frequency of consumption				
	< 8	60·1 %	≥ 8	39·9 %	*P* value*	≤1 drink/week		>1 drink/week		*P* value*
	*n*	%	*n*	%		*n*	%	*n*	%	
Maternal education level										
High† (52·2 %)	1198	60·7 %	776	39·3 %	0·535	1460	77·1 %	433	22·9 %	< 0·001
Low‡ (47·8 %)	1080	59·7 %	729	40·3 %		1236	71·5 %	492	28·5 %	
Household socio-economic status										
High§ (79·4 %)	1749	60·6 %	1139	39·4 %	0·810	2035	73·7 %	725	26·3 %	0·010
Low‖ (20·6 %)	450	60·1 %	299	39·9 %		566	78·4 %	156	21·6 %	
Ethnicity										
White (97·8 %)	2204	59·8 %	1483	40·2 %	0·0004	2629	74·3 %	909	25·7 %	0·013
Non-White (2·1 %)	64	75·3 %	21	24·7 %		60	82·2 %	13	17·8 %	
Maternal AUDIT score										
< 8 (53·0 %)	734	66·1 %	373	33·9 %	< 0·001	830	78·9 %	222	21·1 %	< 0·001
≥ 8 (47·0 %)	522	52·9	464	47·1 %		650	67·4 %	315	32·6 %	
Gender										
Male (43·9 %)	1062	58·3 %	760	41·7 %	0·036	1212	69·5 %	533	30·5 %	< 0·001
Female (56·1 %)	1430	61·5 %	895	38·5 %		1745	78·5 %	478	21·5 %	
Dietary pattern scores at 3 years of age										
Processed										
Mean	−0·24		−0·16		0·023	−0·18		−0·27		0·009
sd	0·90		0·92			0·92		0·89		
Healthy										
Mean	0·05		0·09		0·253	0·03		0·17		< 0·001
sd	0·98		1·01			0·97		1·03		
Traditional										
Mean	0·03		−0·02		0·161	0·01		0·01		0·946
sd	1·00		1·02			1·00		1·01		
Snacks										
Mean	0·01		0·10		0·986	0·09		0·12		0·451
sd	0·94		0·98			0·95		0·97		
Dietary pattern scores at 7 years of age										
Processed										
Mean	−0·16		−0·12		0·336	−0·14		−0·16		0·494
sd	0·95		0·90			-0·17		0·92		
Healthy										
Mean	0·04		0·08		0·291	0·01		0·19		<0·001
sd	0·99		1·01			0·97		1·07		
Traditional										
Mean	0·09		−0·03		0·075	0·17		−0·01		0·484
sd	0·96		0·95			0·96		0·95		
Sugar % overall energy intake at 3 years of age										
Mean	14·16		14·43		0·053	14·31		14·17		0·344
sd	3·95		3·97			3·97		3·73		
Sugar % overall energy intake at 7 years of age										
Mean	14·02		14·25		0·070	14·14		14·17		0·793
sd	3·54		3·40			3·54		3·33		

AUDIT, Alcohol Use Disorders Identification Test.

* *P*-values from *χ* ^2^ tests for categorical variables and *t*-tests for continuous variables.

† Degree or A levels ((optional) exams taken at the age of 18 years).

‡ GCSE/O levels (compulsory exams taken at the age of 16 years) or vocational qualifications.

§ Classes I, II, III (non-manual): professional, managerial/technical or skilled non-manual occupations.

‖ Classes III (manual), IV, V: skilled manual, partly skilled or unskilled occupations.

There were no differences in mean dietary pattern scores at 3 or 7 years of age with AUDIT score. However, children consuming more than one alcoholic drink per week had lower mean scores for the ‘processed’ dietary pattern at 3 and higher mean scores for the ‘healthy’ dietary pattern at both 3 and 7. Sugar intake was slightly higher in those children with an AUDIT score ≥ 8 (although this was < 0·5 %), but there was no difference in mean sugar intake according to frequency of alcohol consumption.

Supplemental Table A outlines the baseline differences in participants who did and did not provide alcohol consumption data at the age of 17 years. Mean maternal age at delivery was 1·3 years higher on average in mothers of participants who provided alcohol data. Participants who provided alcohol data were also more likely to have mothers with a higher level of education and to be from a higher social class, as well as less likely to have mothers with an AUDIT score of ≥ 8. Following multiple imputation, the descriptive characteristics of the participants who did and did not have alcohol data were similar to the non-imputed data (see online Supplemental Table A).

### Dietary patterns and harmful alcohol consumption

Table 2 presents the associations between dietary pattern scores at 3 and 7 years of age and AUDIT scores ≥ 8 (i.e. harmful alcohol consumption). Children in the highest quintile for the 3-year ‘processed’ pattern were more likely to have harmful alcohol drinking compared to those in the lowest quintile (unadjusted OR: 1·41 (95 % CI 1·13, 1·77)). This association was attenuated after adjustment (OR: 1·35 (95 % CI 0·96, 1·90)). A linear association was also evident with an unadjusted OR of 1·09 (95 % CI 1·00, 1·17), but again this was attenuated after adjustment (OR: 1·06 (95 % CI 0·94, 1·19)). There were no associations evident between the ‘processed’ pattern at 7 years and harmful alcohol drinking. For the ‘healthy’ dietary pattern, there was a linear adjusted effect at the age of 7 years (OR: 1·10 (95 % CI 1·00, 1·21)) but not at 3 years of age. The ‘traditional’ pattern showed a protective linear adjusted effect at the age of 3 years (OR; 0·90 (95 % CI 0·82, 1·00)) and this was stronger at the age of 7 years (OR: 0·87 (95 % CI 0·70, 0·96)). In addition, being in the highest quintile of for the ‘traditional’ pattern was associated with a reduced risk of harmful alcohol consumption (OR of 0·71 (95 % CI 0·53, 0·96) and 0·64 (95 % CI 0·47, 0·87) at the age of 3 and 7 years, respectively). The 3-year ‘snack’ pattern was not associated with harmful alcohol consumption. In the imputed analyses presented in Supplemental Table B, similar patterns of association were seen across all dietary patterns, although associations tended to be weaker. This was particularly the case for the ‘traditional’ pattern at the age of 7 years (adjusted OR: 0·94 (95 % CI 0·88, 1·00).

**Table 2:** Association between dietary patterns at the age of 3 and 7 years, and AUDIT score of 8 or greater at the age of 17 years; associations with quintiles of dietary pattern score and continuous pattern score

Exposure	Unadjusted OR	95 % CI	*P* value	Adjusted OR	95 % CI*	*P* value
‘Processed’ diet pattern – 3 years of age						
Number of cases/total	1393/3520			764/1935		
Quintile 1 (baseline – lowest ‘processed’ pattern)	1			1		
Quintile 2	1·32	1·08, 1·60	0·006	1·26	0·98, 1·62	0·077
Quintile 3	1·21	0·98, 1·48	0·070	1·12	0·85, 1·46	0·431
Quintile 4	1·10	0·89, 1·36	0·399	0·96	0·71, 1·30	0·776
Quintile 5 (highest ‘processed’ pattern)	1·41	1·13, 1·77	0·003	1·35	0·96, 1·90	0·083
Linear effect	1·09	1·00, 1·17	0·023	1·06	0·94, 1·19	0·353
‘Processed’ diet pattern – 7 years of age						
Number of cases/total	1323/3311			759/1905		
Quintile 1 (baseline – lowest ‘processed’ pattern)	1			1		
Quintile 2	1·15	0·93, 1·41	0·193	1·17	0·90, 1·53	0·237
Quintile 3	1·05	0·85, 1·30	0·667	0·92	0·69, 1·22	0·541
Quintile 4	1·31	1·06, 1·62	0·013	1·25	0·94, 1·67	0·131
Quintile 5 (highest ‘processed’ pattern)	1·07	0·85, 1·35	0·564	1·09	0·79, 1·50	0·618
Linear effect	1·04	0·96, 1·12	0·336	1·03	0·92, 1·15	0·599
‘Healthy’ diet pattern – 3 years of age						
Number of cases/total	1393/3520			764/1935		
Quintile 1 (baseline – lowest ‘healthy’ pattern)	1			1		
Quintile 2	1·13	0·90, 1·41	0·296	1·26	0·92, 1·73	0·151
Quintile 3	1·07	0·86, 1·34	0·526	1·27	0·94, 1·73	0·122
Quintile 4	1·08	0·87, 1·35	0·475	1·13	0·83, 1·55	0·435
Quintile 5 (highest ‘healthy’ pattern)	1·18	0·95, 1·47	0·129	1·32	0·97, 1·80	0·079
Linear effect	1·04	0·97, 1·11	0·253	1·08	0·98, 1·19	0·143
‘Healthy’ diet pattern – 7 years of age						
Number of cases/total	1323/3311			759/1905		
Quintile 1 (baseline – lowest ‘healthy’ pattern)	1			1		
Quintile 2	0·98	0·78, 1·23	0·870	0·99	0·72, 1·35	0·930
Quintile 3	0·74	0·59, 0·93	0·010	0·79	0·58, 1·08	0·142
Quintile 4	0·85	0·68, 1·07	0·165	0·79	0·58, 1·08	0·144
Quintile 5 (highest ‘healthy’ pattern)	1·09	0·87, 1·36	0·452	1·14	0·83, 1·55	0·420
Linear effect	1·04	0·97, 1·11	0·291	1·10	1·00, 1·21	0·063
‘Traditional’ diet pattern – 3 years of age						
Number of cases/total	1393/3520			764/1935		
Quintile 1 (baseline – lowest ‘traditional’ pattern)	1			1		
Quintile 2	0·99	0·80, 1·22	0·892	0·78	0·59, 1·05	0·100
Quintile 3	0·95	0·77, 1·18	0·642	0·80	0·59, 1·07	0·137
Quintile 4	0·87	0·70, 1·08	0·205	0·73	0·54, 0·97	0·031
Quintile 5 (highest ‘traditional’ pattern)	0·89	0·72, 1·10	0·290	0·71	0·53, 0·96	0·024
Linear effect	0·95	0·89, 1·01	0·161	0·90	0·82, 1·00	0·034
‘Traditional’ diet pattern – 7 years of age						
Number of cases/total	1323/3311			759/1905		
Quintile 1 (baseline – lowest ‘traditional’ pattern)	1			1		
Quintile 2	0·87	0·70, 1·09	0·223	0·78	0·58, 1·05	0·105
Quintile 3	0·96	0·77, 1·20	0·717	0·83	0·62, 1·12	0·220
Quintile 4	0·92	0·74, 1·15	0·448	0·84	0·63, 1·13	0·254
Quintile 5 (highest ‘traditional’ pattern)	0·75	0·60, 0·94	0·011	0·64	0·47, 0·87	0·005
Linear effect	0·94	0·87, 1·01	0·075	0·87	0·70, 0·96	0·007
‘Snack’ diet pattern – 3 years of age						
Number of cases/total	1393/3520			764/1935		
Quintile 1 (baseline – lowest ‘traditional’ pattern)	1			1		
Quintile 2	0·78	0·62, 0·99	0·037	0·74	0·53, 1·03	0·073
Quintile 3	0·81	0·64, 1·01	0·065	0·79	0·58, 1·09	0·154
Quintile 4	0·84	0·67, 1·05	0·123	0·81	0·59, 1·11	0·190
Quintile 5 (highest ‘traditional’ pattern)	0·91	0·73, 1·14	0·407	0·87	0·64, 1·19	0·390
Linear effect	0·99	0·93, 1·07	0·986	0·97	0·88, 1·07	0·572

AUDIT, Alcohol Use Disorders Identification Test.

* Data adjusted for ethnicity, maternal level of education, parental social class and maternal AUDIT score.

### Dietary patterns and frequent alcohol consumption

Associations between dietary pattern scores and whether participants consumed more than one alcoholic drink per week at the age of 17 years are presented in Table 3. Being in the highest quintile of the ‘processed’ pattern score at the age of 7 years was protective (OR: 0·70 (95 % CI 0·54, 0·91)) compared to being in the lowest quintile; however, this association was lost after adjustment for confounders. Similarly, the unadjusted linear association with the ‘processed’ pattern scores pre-adjustment was no longer evident after adjustment. The 7-year ‘processed’ pattern was not associated with consuming more than one alcoholic drink per week at the age of 17 years. The ‘healthy’ pattern at both 3 and 7 years of age was linearly associated with this outcome after adjustment, although the effects were stronger at 7 years of age compared to 3 years of age (OR: 1·21 and 1·11, respectively). There were no associations between the ‘traditional’ dietary pattern at either age or the ‘snacks’ pattern at the age of 3 years and later alcohol consumption at the age of 17 years. All these patterns of association were replicated in the imputed analyses (see online Supplemental Table C). There was evidence of an interaction between gender and the unadjusted 3-year ‘processed’ pattern and the adjusted 7-year ‘traditional’ pattern. Additional stratified analyses were therefore carried out (see online Supplemental Table E). This indicated that boys with the highest ‘processed’ dietary pattern quintile were 0·55 (95 % CI 0·37, 0·80) times less likely to consume alcohol, whilst girls in the same quintile had no association with an OR of 0·92 (95 % CI 0·64, 1·33). No association was shown for girls or boys separately for the ‘traditional’ pattern.

**Table 3:** Association between dietary patterns at ages 3 and 7 years, and consumption of more than one drink per week at the age of 17 years; associations with quintiles of dietary pattern score and continuous pattern score

Exposure	Unadjusted OR	95 % CI	*P* value	Adjusted OR	95 % CI	*P* value
‘Processed’ diet pattern – 3 years of age						
Number of cases/total	860/3371			492/1856		
Quintile 1 (baseline – lowest ‘processed’ pattern)	1			1		
Quintile 2	0·90	0·72, 1·12	0·340	0·83	0·62, 1·10	0·199
Quintile 3	0·97	0·78. 1·22	0·805	1·00	0·75, 1·35	0·976
Quintile 4	0·74	0·58, 0·95	0·018	0·72	0·50, 1·02	0·061
Quintile 5 (highest ‘processed’ pattern)	0·70	0·54, 0·91	0·009	0·90	0·61, 1·32	0·597
Linear effect	0·89	0·82, 0·97	0·009	0·94	0·82, 1·08	0·376
‘Processed’ diet pattern – 7 years of age						
Number of cases/total	809/3175			485/1832		
Quintile 1 (baseline – lowest ‘processed’ pattern)	1			1		
Quintile 2	0·94	0·75, 1·19	0·621	0·83	0·61, 1·12	0·221
Quintile 3	0·92	0·72, 1·17	0·508	0·86	0·62, 1·17	0·332
Quintile 4	0·99	0·78, 1·27	0·965	1·08	0·79, 1·48	0·638
Quintile 5 (highest ‘processed’ pattern)	0·80	0·61, 1·05	0·102	0·76	0·52, 1·10	0·147
Linear effect	0·97	0·89, 1·06	0·494	1·00	0·89, 1·14	0·960
‘Healthy’ diet pattern – 3 years of age						
Number of cases/total	860/3371			492/1856		
Quintile 1 (baseline – lowest ‘healthy’ pattern)	1			1		
Quintile 2	0·87	0·67, 1·14	0·308	0·80	0·55, 1·15	0·221
Quintile 3	1·09	0·84, 1·41	0·520	0·83	0·59, 1·18	0·311
Quintile 4	1·21	0·94, 1·56	0·137	1·05	0·75, 1·49	0·768
Quintile 5 (highest ‘healthy’ pattern)	1·37	1·07, 1·75	0·013	1·13	0·80, 1·58	0·494
Linear effect	1·15	1·06, 1·23	<0·001	1·11	1·00, 1·24	0·056
‘Healthy’ diet pattern – 7 years of age						
Number of cases/total	809/3175			485/1832		
Quintile 1 (baseline – lowest ‘healthy’ pattern)	1			1		
Quintile 2	1·03	0·78, 1·34	0·854	0·96	0·67, 1·37	0·813
Quintile 3	0·82	0·62, 1·08	0·156	0·66	0·45, 0·96	0·028
Quintile 4	1·13	0·87, 1·47	0·352	0·95	0·66, 1·35	0·763
Quintile 5 (highest ‘healthy’ pattern)	1·52	1·18, 1·96	0·001	1·35	0·95, 1·91	0·093
Linear effect	1·19	1·10, 1·29	<0·001	1·21	1·09, 1·35	0·001
‘Traditional’ diet pattern – 3 years of age						
Number of cases/total	860/3371			492/1856		
Quintile 1 (baseline – lowest ‘traditional’ pattern)	1			1		
Quintile 2	1·21	0·95, 1·57	0·120	1·10	0·79, 1·52	0·576
Quintile 3	0·97	0·75, 1·25	0·811	0·95	0·68, 1·33	0·776
Quintile 4	1·05	0·82, 1·34	0·718	0·97	0·70, 1·35	0·874
Quintile 5 (highest ‘traditional’ pattern)	1·02	0·80, 1·30	0·884	0·88	0·63, 1·24	0·469
Linear effect	1·00	0·92, 1·08	0·946	0·95	0·85, 1·05	0·312
‘Traditional’ diet pattern – 7 years of age						
Number of cases/total	809/3175			485/1832		
Quintile 1 (baseline – lowest ‘traditional’ pattern)	1			1		
Quintile 2	0·89	0·69, 1·15	0·384	0·94	0·67, 1·32	0·736
Quintile 3	1·00	0·78, 1·29	0·979	1·02	0·73, 1·43	0·899
Quintile 4	0·93	0·72, 1·20	0·598	0·97	0·70, 1·36	0·880
Quintile 5 (highest ‘traditional’ pattern)	0·89	0·69, 1·15	0·375	0·89	0·63, 1·26	0·506
Linear effect	0·97	0·89, 1·06	0·484	0·98	0·87, 1·09	0·663
‘Snack’ diet pattern – 3 years of age						
Number of cases/total	1393/3520			764/1935		
Quintile 1 (baseline – lowest ‘traditional’ pattern)	1			1		
Quintile 2	0·85	0·65, 1·10	0·205	0·77	0·53, 1·12	0·169
Quintile 3	0·83	0·64, 1·08	0·160	0·88	0·62, 1·26	0·481
Quintile 4	0·90	0·70, 1·16	0·424	0·82	0·58, 1·17	0·275
Quintile 5 (highest ‘traditional’ pattern)	1·04	0·81, 1·34	0·765	1·02	0·71, 1·45	0·928
Linear effect	1·03	0·95, 1·11	0·450	1·01	0·91, 1·14	0·804

### Sugar consumption and alcohol consumption

There was an increased risk of harmful alcohol consumption for those children in the highest quintile of sugar intake at the age of 3 years, but the OR was attenuated after adjustment (Table 4), and there were no associations with sugar intake at the age of 7 years. There were no associations between sugar consumption at either age and having more than one alcoholic drink per week at 17 years of age. In the imputed analysis (see online Supplemental Table E), a similar pattern was seen.

**Table 4:** Association between percentage of overall energy intake as NMES at the age of 3 and 7 years, and alcohol consumption at the age of 17 years; associations with quintiles of NMES and continuous NMES intake

Outcome 1 – AUDIT score of 8 or greater at the age of 17 years						
Exposure	Unadjusted OR	95 % CI	*P* value	Adjusted OR	95 % CI	*P* value
Sugar % overall energy intake – 3 years of age						
Number of cases/total	1381/3472			784/1917		
Quintile 1 (baseline – lowest sugar)	1			1		
Quintile 2	0·94	0·77, 1·16	0·588	1·09	0·82, 1·44	0·566
Quintile 3	1·07	0·87, 133	0·502	1·08	0·81, 1·45	0·580
Quintile 4	1·01	0·82, 1·25	0·914	1·05	0·78, 1·42	0·726
Quintile 5 (highest sugar)	1·24	0·99, 1·54	0·057	1·30	0·95, 1·78	0·105
Linear effect	1·02	1·00, 1·03	0·053	1·01	0·98, 1·04	0·309
Sugar % overall energy intake – 7 years of age						
Number of cases/total	1975/3288			757/1898		
Quintile 1 (baseline – lowest sugar)	1			1		
Quintile 2	1·21	0·97, 1·50	0·084	1·09	0·83, 1·44	0·526
Quintile 3	1·33	1·07, 1·65	0·009	1·25	0·94, 1·66	0·123
Quintile 4	1·24	0·99, 1·54	0·056	0·99	0·74, 1·32	0·926
Quintile 5 (highest sugar)	1·19	0·95, 1·50	0·139	1·04	0·76, 1·42	0·792
Linear effect	1·02	0·99, 1·04	0·070	1·00	0·97, 1·03	0·927
Outcome 2 – more than one drink per week; associations with quintiles of NMES and continuous NMES intake						
Sugar % overall energy intake – 3 years of age						
Number of cases/total	853/3332			491/1841		
Quintile 1 (baseline – lowest sugar)	1			1		
Quintile 2	0·89	0·70, 1·13	0·351	0·88	0·64, 1·20	0·417
Quintile 3	1·05	0·83, 1·33	0·684	1·06	0·77, 1·46	0·730
Quintile 4	1·03	0·81, 1·31	0·836	1·06	0·76, 1·47	0·720
Quintile 5 (highest sugar)	0·83	0·64, 1·08	0·170	1·02	0·71, 1·46	0·915
Linear effect	0·99	0·97, 1·01	0·344	1·01	0·98, 1·04	0·608
Sugar % overall energy intake – 7 years of age						
Number of cases/total	804/3148			484/1824		
Quintile 1 (baseline – lowest sugar)	1			1		
Quintile 2	1·06	0·83, 1·36	0·618	0·97	0·71, 1·32	0·837
Quintile 3	1·06	0·83, 1·36	0·656	0·91	0·66, 1·26	0·580
Quintile 4	1·09	0·85, 1·40	0·503	0·97	0·70, 1·35	0·876
Quintile 5 (highest sugar)	1·06	0·82, 1·38	0·645	1·03	0·73, 1·46	0·853
Linear effect	1·00	0·98, 1·03	0·793	1·00	0·77, 1·04	0·831

NMES, non-milk extrinsic sugar; AUDIT, Alcohol Use Disorders Identification Test.

## Discussion

In this study, we report an association between a ‘healthy’ dietary pattern score at both 3 and 7 years of age and consuming more than one alcohol drink per week at the age of 17 years. In addition, the ‘traditional’ dietary pattern at both ages was protective of harmful alcohol intake at the age of 17 years. However, we found no association between sugar intake and either measure of alcohol consumption after adjustment for ethnicity, maternal level of education, parental social class and maternal AUDIT score.

This study adds to a limited and new area of literature. Nutrition in childhood has been shown to impact on a wide range of later health and behavioural outcomes^(32,33)^. Given the negative impact that alcohol can have on health, exploration of factors that contribute to increased usage is important. It is reassuring that in this population, a poor diet in childhood (represented by adherence to the ‘processed’ pattern) is not associated with an increased risk of harmful drinking in early adulthood. The fact that adhering to a ‘healthy’ pattern in early childhood is associated with an increased likelihood of consuming more than one alcoholic drink a week may warrant further investigation. However, harmful levels of intake were not associated with this dietary pattern.

We are not aware of any other studies examining the association between dietary patterns in childhood and alcohol consumption in adolescence. However, other studies have identified associations between dietary patterns and alcohol consumption in adulthood which helped to inform our hypothesis. For example, a positive association was identified between heavier alcohol consumption during pregnancy and scoring highly on the ‘processed’ dietary pattern among mothers in the ALSPAC cohort^(34)^. The FinDrink project in Finland identified mixed results from a study of the relationship between alcohol consumption and dietary patterns^(35)^, and they identified that moderate alcohol consumption (compared to non-drinkers) was identified with higher fish intake but also higher energy intake from total fats and monosaturated fats. A key conclusion from the FinDrink study was that further study into the relationship between alcohol consumption and dietary habits is needed.

The study by Mehlig *et al.* also informed our hypothesis, and specifically our interest in examining the relationship between NMES percentage intake and alcohol consumption. The results from the ALSPAC study are notably different to the IDEFICS study and could be due to multiple factors. Firstly, there are likely to be differences between the demographics, eating habits and alcohol use of the populations from different countries^(12–14)^. Secondly, the ALSPAC data offered the opportunity to study a larger population than the IDEFICS study. Approximately 25·5 % of the adolescents were consuming at least one drink per week and 39·9 % had an AUDIT => 8, which provided a rich data source for identifying if any associations exist. This contrasts with the study undertaken by Mehlig *et al.* ^(10)^ which had 107 participants regularly consuming alcohol. This is a small sample size when considering that the results are spread across eight countries. A further strength of the ALSPAC dataset is that the study participants were 17 years of age. In the UK, the legal age for alcohol consumption is 18 years; however, by the age of 17 years, most adolescents have tried alcohol^(6)^ and could have developed patterns of drinking which can be assessed using the AUDIT scoring system^(36)^. In IDEFICS, the adolescents were aged 11–16 years^(10)^, so it is more unlikely that this cohort would be regularly consuming alcohol^(6)^. Reporting of alcohol intake may have been affected as the participants were underage. The use of AUDIT scores also strengthens our study. AUDIT is a widely used, validated tool^(25)^, and the overall score offers a broad understanding of a person’s risk of alcohol harm. To add sensitivity to the study, the measure of alcohol frequency was included as an additional outcome variable. This additional measure also had more similarity to the outcome measure in the IDEFICS study, providing an opportunity to better consider our paper in the context of previous findings. Finally, it should be highlighted that the IDEFICS study concluded that both sugar and fat intake were associated with later alcohol consumption. High fat intake was shown to be independently associated with alcohol consumption, but high sugar intake was not^(10)^. Our findings support this lack of independent association with sugar; however, we did not find any evidence to suggest that increased ‘fat’ intake, using our ‘processed’ pattern as a proxy was associated with alcohol consumption.

As well as the strengths outlined above, this paper has a number of limitations. Some children were lost to follow-up, reducing the amount of data available at the later time points. However, the results of the imputation indicated that this did not bias our findings. On the whole, effect sizes in the imputed analyses were smaller than those in the complete case analysis. The loss of participants to follow-up reduces the external validity of the study, because the adolescents included were more likely to be from advantaged backgrounds, more likely to be from a White British background, more likely to have highly educated mothers and less likely to have mothers with an AUDIT => 8.

A further potential limitation is the reliability of self-reported alcohol data amongst adolescents^(37,38)^. Williams *et al.* ^(37)^ have shown that self-report of substance abuse by adolescents only had fair validity and recommended biochemical corroboration be routinely used for this age group, although Winters *et al.* ^(38)^ suggest that adolescents reliably self-report alcohol intake. The data collected on alcohol were part of a much wider clinic visit with many other measures being obtained. Whilst there is still a risk that participants may have under-reported their alcohol consumption, it is less likely in such a setting where multiple facets of an individual are being examined.

The external validity of the study may also be affected by the changes in children’s diets over the last 30 years. The dietary intake data used in the study were collected in the 1990s, and between data collection and the present day, childhood obesity has increased and some elements of childhood diet have changed in association with this^(39)^. It is likely that the broad patterns found in the PCA would remain, but some loadings and scores that contributed to the patterns may have strengthened, for example, to accommodate an increase in consumption of sugary drinks^(40)^.

## Conclusion

In this large prospective cohort study, no association was found between sugar intake in childhood and alcohol consumption in later adolescence, which is in contradiction to previous research on this topic. However, we do report an association between adherence to a ‘healthy’ dietary pattern at both 3 and 7 years of age and consuming more than one drink per week and adherence to a ‘traditional’ dietary pattern intake at the age of 7 years being protective of harmful alcohol intake. We do not know enough from this study alone to know if adherence to a specific dietary pattern in early childhood would affect harm from alcohol. However, the study adds to a field of relatively limited literature and further research is required to elucidate the associations under study. For the population studied, it suggests that changes to diet in early childhood are unlikely to have an impact on harmful alcohol use in adolescence given the lack of consistency across the results.

## Supplementary material

For supplementary material accompanying this paper visit https://doi.org/10.1017/S1368980021004183. click here to view supplementary material
