# Supplementary material for: Are dietary patterns in early childhood associated with alcohol consumption at the age of 17 years? Analysis of data from the Avon Longitudinal Study of Parents and Children (ALSPAC) prospective cohort study
Source: Public Health Nutr. 2021 Oct 6;25(9):2488–97. doi: 10.1017/S1368980021004183 (PMC9991802; doi:10.1017/S1368980021004183)
Supplement: Supplementary file 1 [file S1368980021004183sup001.docx]

**Supplementary Table A** Baseline characteristics of children in the imputed dataset and those with alcohol data available at age 17 (basis for complete case analysis); n(%) for categorial variables, mean(sd) for continuous variables

|  | **Imputed data** | **Sample with alcohol data available (n=4148)** |
| --- | --- | --- |
| **Maternal age (years) at delivery** | 28.0 (4.96) | 29.3 (4.57) |
| **Maternal education level**  High^b^  Low^c^ | 34.3%  65.7% | 35.3%  64.7% |
| **Household socio-economic status**  Highd  Lowe | 29.3%  70.7% | 27.2%  72.8% |
| **Ethnicity**  White  Non-white | 97.3%  2.7% | 97.8%  2.2% |
| **Maternal AUDIT score**  < 8  ≥ 8 | 59.3%  40.7% | 60.1%  39.9% |
| **Gender**  Male  Female | 51.7%  48.3% | 51.2%  48.3% |

*^a^ t-test for continuous data or chi-squared for categorical data*

*^b^ Degree or A levels ((optional) exams taken at the age of 18);*

*^c^ GCSEs/O levels (compulsory exams taken at the age of 16) or vocational qualifications*

*^d^ classes I, II, III (non-manual): Professional, managerial/technical or skilled non-manual occupations*

*^e^ classes III (manual), IV, V: Skilled manual, partly-skilled or unskilled occupations*

**Supplementary Table B** Association between dietary patterns at ages 3 and 7, and AUDIT score of 8 or greater at age 17 in imputed data (n=13966, those with AUDIT score of 8 or greater= 5601) ; associations with quintiles of dietary pattern score and continuous pattern score

| **Exposure** | | **Unadjusted OR (95% CI)** | | **P value** | | **Adjusted OR (95% CI)^[[1]](#footnote-1)^** | | **P value** | |
| --- | --- | --- | --- | --- | --- | --- | --- | --- | --- |
| **“Processed” diet pattern – age** | |  | |  | |  | | | |
| Quintile 1 (baseline - lowest “processed” pattern) | | 1 | |  | | 1 | |  | |
| Quintile 2 | | 1.12 (0.95; 1.31) | | 0.172 | | 1.12 (0.95; 1.32) | | 0.164 | |
| Quintile 3 | | 1.13 (0.97, 1.31) | | 0.112 | | 1.13 (0.96, 1.33) | | 0.128 | |
| Quintile 4 | | 1.15 (0.95, 1.40) | | 0.149 | | 1.16 (0.95, 1.41) | | 0.140 | |
| Quintile 5 (highest “processed” pattern) | | 1.31 (1.03, 1.68) | | 0.031 | | 1.30 (1.01, 1.68) | | 0.040 | |
| Linear effect | | 1.08 (1.01, 1.17) | | 0.034 | | 1.08 (1.00, 1.17) | | 0.047 | |
| **“Processed” diet pattern – age 7** | |  | |  | |  | | | |
| Quintile 1 (baseline - lowest “processed” pattern) | | 1 | |  | | 1 | |  | |
| Quintile 2 | | 1.06 (0.91; 1.22) | | 0.466 | | 1.04 (0.89; 1.21) | | 0.584 | |
| Quintile 3 | | 1.08 (0.91, 1.29) | | 0.395 | | 1.05 (0.88, 1.25) | | 0.620 | |
| Quintile 4 | | 1.17 (0.97, 1.42) | | 0.100 | | 1.13 (0.93, 1.37) | | 0.207 | |
| Quintile 5 (highest “processed” pattern) | | 1.19 (0.96, 1.49) | | 0.115 | | 1.13 (0.91, 1.41) | | 0.274 | |
| Linear effect | | 1.06 (0.98, 1.14) | | 0.127 | | 1.04 (0.97, 1.13) | | 0.252 | |
|  | |  | |  | |  | |  | |
| **“Healthy” diet pattern – age 3** | |  | |  | |  | | | |
| Quintile 1 (baseline - lowest “healthy” pattern) | | 1 | |  | | 1 | |  | |
| Quintile 2 | | 1.04 (0.90; 1.21) | | 0.593 | | 1.05 (0.91; 1.22) | | 0.495 | |
| Quintile 3 | | 1.04 (0.87, 1.23) | | 0.671 | | 1.05 (0.89, 1.26) | | 0.550 | |
| Quintile 4 | | 1.07 (0.87, 1.30) | | 0.530 | | 1.09 (0.88, 1.36) | | 0.426 | |
| Quintile 5 (highest “healthy” pattern) | | 1.10 (0.89, 1.37) | | 0.381 | | 1.15 (0.91, 1.47) | | 0.239 | |
| Linear effect | | 1.03 (0.96, 1.11) | | 0.440 | | 1.04 (0.96, 1.12) | | 0.366 | |
| **“Healthy” diet pattern – age 7** | |  | |  | |  | | | |
| Quintile 1 (baseline - lowest “healthy” pattern) | | 1 | |  | | 1 | |  | |
| Quintile 2 | | 1.00 (0.86; 1.16) | | 0.988 | | 1.00 (0.87; 1.16) | | 0.988 | |
| Quintile 3 | | 0.92 (0.77, 1.10) | | 0.379 | | 0.93 (0.77, 1.11) | | 0.420 | |
| Quintile 4 | | 0.96 (0.81, 1.15) | | 0.693 | | 0.98 (0.81, 1.17) | | 0.799 | |
| Quintile 5 (highest “healthy” pattern) | | 1.04 (0.83, 1.29) | | 0.749 | | 1.06 (0.85, 1.32) | | 0.626 | |
| Linear effect | | 1.02 (0.95, 1.09) | | 0.446 | | 1.02 (0.96, 1.11) | | 0.446 | |
|  | |  | |  | |  | |  | |
| **“Traditional” diet pattern – age 3** | |  | |  | |  | | | |
| Quintile 1 (baseline - lowest “traditional” pattern) | | 1 | |  | | 1 | |  | |
| Quintile 2 | | 0.97 (0.85; 1.11) | | 0.705 | | 0.97 (0.85; 1.11) | | 0.667 | |
| Quintile 3 | | 0.95 (0.82, 1.09) | | 0.452 | | 0.96 (0.83, 1.10) | | 0.542 | |
| Quintile 4 | | 0.91 (0.75, 1.09) | | 0.303 | | 0.92 (0.76, 1.12) | | 0.412 | |
| Quintile 5 (highest “traditional” pattern) | | 0.90 (0.74, 1.08) | | 0.258 | | 0.92 (0.76, 1.12) | | 0.401 | |
| Linear effect | | 0.96 (0.91, 1.02) | | 0.188 | | 0.96 (0.91, 1.03) | | 0.254 | |
| **“Traditional” diet pattern – age 7** | |  | |  | |  | | | |
| Quintile 1 (baseline - lowest “traditional” pattern) | | 1 | |  | | 1 | |  | |
| Quintile 2 | | 0.94 (0.81; 1.09) | | 0.407 | | 0.94 (0.81; 1.09) | | 0.437 | |
| Quintile 3 | | 0.91 (0.77, 1.07) | | 0.245 | | 0.91 (0.77, 1.07) | | 0.268 | |
| Quintile 4 | | 0.89 (0.75, 1.05) | | 0.170 | | 0.90 (0.76, 1.07) | | 0.225 | |
| Quintile 5 (highest “traditional” pattern) | | 0.82 (0.67, 1.00) | | 0.048 | | 0.83 (0.68, 1.01) | | 0.068 | |
| Linear effect | | 0.94 (0.88, 1.00) | | 0.059 | | 0.94 (0.88, 1.00) | | 0.061 | |
| **“Snack” diet pattern – age 3** |  | |  | |  | | | |  |
| Quintile 1 (baseline - lowest “traditional” pattern) | 1 | |  | | 1 | |  | |  |
| Quintile 2 | 0.93 (0.81, 1.08) | | 0.358 | | 0.92 (0.80, 1.07) | | 0.289 | |  |
| Quintile 3 | 0.92 (0.77, 1.11) | | 0.398 | | 0.92 (0.76, 1.10) | | 0.349 | |  |
| Quintile 4 | 0.92 (0.75, 1.14) | | 0.450 | | 0.91 (0.74, 1.11) | | 0.371 | |  |
| Quintile 5 (highest “traditional” pattern) | 0.95 (0.75, 1.20) | | 0.679 | | 0.93 (0.74, 1.17) | | 0.537 | |  |
| Linear effect | 0.99 (0.91, 1.07) | | 0.760 | | 0.98 (0.90, 1.07) | | 0.678 | |  |

**Supplementary Table C** Association between dietary patterns at ages 3 and 7, and consumption of more than one drink per week at age 17 in imputed data (n=13966, those consuming more than one drink per week=949) ; associations with quintiles of dietary pattern score and continuous pattern score

| **Exposure** | **Unadjusted OR (95% CI)** | **P value** | **Adjusted OR (95% CI)** | **P value** |  |
| --- | --- | --- | --- | --- | --- |
| **“Processed” diet pattern – age 3** |  |  |  | |  |
| Quintile 1 (baseline - lowest “processed” pattern) | 1 |  | 1 |  |  |
| Quintile 2 | 0.90 (0.73; 1.12) | 0.358 | 0.93 (0.74; 1.16) | 0.507 |  |
| Quintile 3 | 0.96 (0.77, 1.21) | 0.731 | 1.00 (0.79, 1.27) | 0.988 |  |
| Quintile 4 | 0.75 (0.59, 0.95) | 0.018 | 0.81 (0.62, 1.04) | 0.097 |  |
| Quintile 5 (highest “processed” pattern) | 0.70 (0.54, 0.91) | 0.008 | 0.80 (0.68, 1.06) | 0.115 |  |
| Linear effect | 0.89 (0.82, 0.97) | 0.009 | 0.93 (0.84, 1.02) | 0.110 | |
| **“Processed” diet pattern – age 7** |  |  |  | |  |
| Quintile 1 (baseline - lowest “processed” pattern) | 1 |  | 1 |  |  |
| Quintile 2 | 0.95 (0.75; 1.20) | 0.669 | 0.94 (0.52; 1.20) | 0.638 |  |
| Quintile 3 | 0.92 (0.73, 1.16) | 0.481 | 0.92 (0.74, 1.17) | 0.497 |  |
| Quintile 4 | 0.97 (0.77, 1.23) | 0.800 | 0.99 (0.72, 1.27) | 0.947 |  |
| Quintile 5 (highest “processed” pattern) | 0.81 (0.63, 1.04) | 0.097 | 0.84 (0.78, 1.09) | 0.194 |  |
| Linear effect | 0.97 (0.89, 1.06) | 0.494 | 0.99 (0.90, 1.09) | 0.843 | |
| **“Healthy” diet pattern – age 3** |  |  |  | |  |
| Quintile 1 (baseline - lowest “healthy” pattern) | 1 |  | 1 |  |  |
| Quintile 2 | 0.86 (0.66; 1.13) | 0.288 | 0.85 (0.65; 1.12) | 0.255 |  |
| Quintile 3 | 1.06 (0.82, 1.36) | 0.651 | 1.04 (0.81, 1.35) | 0.742 |  |
| Quintile 4 | 1.20 (0.93, 1.53) | 0.156 | 1.17 (0.90, 1.51) | 0.242 |  |
| Quintile 5 (highest “healthy” pattern) | 1.34 (1.06, 1.71) | 0.017 | 1.26 (0.98, 1.63) | 0.070 |  |
| Linear effect | 1.15 (1.06, 1.24) | <0.001 | 1.12 (1.04, 1.22) | 0.004 | |
| **“Healthy” diet pattern – age 7** |  |  |  | |  |
| Quintile 1 (baseline - lowest “healthy” pattern) | 1 |  | 1 |  |  |
| Quintile 2 | 1.02 (0.78; 1.35) | 0.869 | 0.99 (0.75; 1.31) | 0.961 |  |
| Quintile 3 | 0.88 (0.66, 1.16) | 0.366 | 0.82 (0.62, 1.09) | 0.177 |  |
| Quintile 4 | 1.16 (0.90, 1.51) | 0.249 | 1.09 (0.83, 1.42) | 0.533 |  |
| Quintile 5 (highest “healthy” pattern) | 1.52 (1.17, 1.96) | 0.002 | 1.37 (1.04, 1.80) | 0.023 |  |
| Linear effect | 1.19 (1.10, 1.27) | <0.001 | 1.17 (1.08, 1.27) | <0.001 | |
| **“Traditional” diet pattern – age 3** |  |  |  | |  |
| Quintile 1 (baseline - lowest “traditional” pattern) | 1 |  | 1 |  |  |
| Quintile 2 | 1.19 (0.93; 1.52) | 0.168 | 1.21 (0.94; 1.56) | 0.132 |  |
| Quintile 3 | 0.97 (0.75, 1.25) | 0.813 | 1.01 (0.79, 1.31) | 0.910 |  |
| Quintile 4 | 1.04 (0.82, 1.33) | 0.740 | 1.11 (0.86, 1.42) | 0.425 |  |
| Quintile 5 (highest “traditional” pattern) | 1.00 (0.79, 1.29) | 0.970 | 1.08 (0.84, 1.39) | 0.536 |  |
| Linear effect | 1.00 (0.92, 1.08) | 0.946 | 1.01 (0.94, 1.09) | 0.777 | |
| **“Traditional” diet pattern – age 7** |  |  |  | |  |
| Quintile 1 (baseline - lowest “traditional” pattern) | 1 |  | 1 |  |  |
| Quintile 2 | 0.90 (0.70; 1.17) | 0.452 | 0.92 (0.71; 1.20) | 0.531 |  |
| Quintile 3 | 0.98 (0.77, 1.26) | 0.891 | 1.00 (0.78, 1.28) | 0.977 |  |
| Quintile 4 | 0.93 (0.72, 1.20) | 0.592 | 0.96 (0.74, 1.24) | 0.763 |  |
| Quintile 5 (highest “traditional” pattern) | 0.90 (0.70, 1.16) | 0.423 | 0.95 (0.74, 1.23) | 0.717 |  |
| Linear effect | 0.97 (0.89, 1.06) | 0.484 | 0.98 (0.90, 1.06) | 0.606 | |
| **“Snack” diet pattern – age 3** |  |  |  |  |  |
| Quintile 1 (baseline - lowest “snack” pattern) | 1 |  | 1 |  |  |
| Quintile 2 | 0.87 (0.66, 1.12) | 0.313 | 0.81 (0.62, 1.06) | 0.122 |  |
| Quintile 3 | 0.86 (0.66, 1.11) | 0.246 | 0.79 (0.61, 1.02) | 0.074 |  |
| Quintile 4 | 0.92 (0.72, 1.18) | 0.532 | 0.83 (0.65, 1.07) | 0.015 |  |
| Quintile 5 (highest “snack” pattern) | 1.06 (0.83, 1.36) | 0.620 | 0.93 (0.72, 1.20) | 0.577 |  |
| Linear effect | 1.03 (0.95, 1.12) | 0.450 | 0.99 (0.91, 1.08) | 0.867 |  |

**Supplementary Table D**: Stratification for variables that showed interaction with gender

*Outcome: Consumption of more than one drink per week^[[2]](#footnote-2)^*

| **“Processed” diet pattern – age 3 (unadjusted)** |  | |  | |  | |  |
| --- | --- | --- | --- | --- | --- | --- | --- |
| Test for interaction (p value) | 0.048 |  | |  | |  | |
| Number of observations | 1498 | |  | |  | |  |
|  |  | |  | |  | |  |
| **Exposure** | **Boys: OR (95% CI)** | | **P value** | | **Girls: OR (95% CI)** | | **P value** |
| Quintile 1 (baseline - lowest “processed” pattern) | 1 | |  | | 1 | |  |
| Quintile 2 | 0.68 (0.50, 0.93) | | 0.016 | | 1.19 (0.87, 1.63) | | 0.277 |
| Quintile 3 | 0.81 (0.58, 1.11) | | 0.184 | | 1.20 (0.87, 1.65) | | 0.279 |
| Quintile 4 | 0.74 (0.53, 1.04) | | 0.088 | | 0.75 (0.52, 1.08) | | 0.127 |
| Quintile 5 (highest “processed” pattern) | 0.55 (0.37, 0.80) | | 0.002 | | 0.92 (0.64, 1.33) | | 0.660 |

| **“Traditional” diet pattern – age 7 (adjusted)** |  |  | |  | |  | |
| --- | --- | --- | --- | --- | --- | --- | --- |
| Test for interaction (p value) | 0.038 |  | |  | |  | |
| Number of observations | 838 | |  | | 994 | |  |
|  |  | |  | |  | |  |
| Quintile 1 (baseline - lowest “traditional” pattern) | 1 | |  | | 1 | |  |
| Quintile 2 | 0.83 (0.51, 1.34) | | 0.449 | | 1.08 (0.66, 1.75) | | 0.766 |
| Quintile 3 | 1.27 (0.79, 2.02) | | 0.321 | | 0.88 (0.53, 1.44) | | 0.609 |
| Quintile 4 | 0.99 (0.62, 1.58) | | 0.978 | | 0.96 (0.59, 1.56) | | 0.885 |
| Quintile 5 (highest “traditional” pattern) | 1.31 (0.81, 2.10) | | 0.266 | | 0.60 (0.35, 1.02) | | 0.057 |

**Supplementary Table E:** Association between percentage of overall energy intake as NMES at ages 3 and 7, and alcohol consumption at 17 in imputed data (n=13966, those with AUDIT score of 8 or greater=5601 and those consuming more than one drink per week = 949); associations with quintiles of NMES and continuous NMES intake

Outcome 1 – AUDIT score of 8 or greater at age 17

| **Exposure** | **Unadjusted OR (95% CI)** | **P value** | **Adjusted OR (95% CI)** | **P value** |  |
| --- | --- | --- | --- | --- | --- |
| **Sugar % overall energy intake – age 3** |  |  |  |  |  |
| Quintile 1 (baseline - lowest sugar) | 1 |  | 1 |  |  |
| Quintile 2 | 1.02 (0.88; 1.19) | 0.758 | 1.03 (0.88; 1.21) | 0.685 |  |
| Quintile 3 | 1.11 (0.94, 1.31) | 0.205 | 1.11 (0.94, 1.31) | 0.226 |  |
| Quintile 4 | 1.12 (0.93, 1.35) | 0.230 | 1.12 (0.93, 1.35) | 0.233 |  |
| Quintile 5 (highest sugar) | 1.26 (1.01, 1.57) | 0.045 | 1.25 (1.00, 1.56) | 0.052 |  |
| Linear effect | 1.02 (1.00, 1.04) | 0.068 | 1.02 (1.00, 1.04) | 0.078 | |
| **Sugar % overall energy intake – age 7** |  |  |  |  |  |
| Quintile 1 (baseline - lowest sugar) | 1 |  | 1 |  |  |
| Quintile 2 | 1.13 (0.96; 1.32) | 0.148 | 1.10 (0.94; 1.29) | 0.212 |  |
| Quintile 3 | 1.17 (0.99, 1.38) | 0.061 | 1.15 (0.98, 1.35) | 0.095 |  |
| Quintile 4 | 1.20 (1.00, 1.44) | 0.051 | 1.16 (0.97, 1.39) | 0.105 |  |
| Quintile 5 (highest sugar) | 1.26 (0.99, 1.61) | 0.055 | 1.22 (0.97, 1.53) | 0.094 |  |
| Linear effect | 1.02 (1.00, 1.04) | 0.053 | 1.02 (1.00, 1.04) | 0.097 | |

Outcome 2 – more than one drink per week

| **Exposure** | **Unadjusted OR (95% CI)** | **P value** | **Adjusted OR (95% CI)** | **P value** |  |
| --- | --- | --- | --- | --- | --- |
| **Sugar % overall energy intake – age 3** |  |  |  |  |  |
| Quintile 1 (baseline - lowest sugar) | 1 |  | 1 |  |  |
| Quintile 2 | 0.90 (0.71; 1.14) | 0.402 | 0.92 (0.72; 1.17) | 0.487 |  |
| Quintile 3 | 1.05 (0.83, 1.32) | 0.690 | 1.07 (0.84, 1.35) | 0.600 |  |
| Quintile 4 | 1.00 (0.79, 1.27) | 0.981 | 1.03 (0.81, 1.30) | 0.808 |  |
| Quintile 5 (highest sugar) | 0.83 (0.64, 1.08) | 0.163 | 0.88 (0.67, 1.15) | 0.359 |  |
| Linear effect | 0.99 (0.97, 1.01) | 0.344 | 0.99 (0.97, 1.02) | 0.612 | |
|  |  |  |  |  |  |
| **Sugar % overall energy intake – age 7** |  |  |  |  |  |
| Quintile 1 (baseline - lowest sugar) | 1 |  | 1 |  |  |
| Quintile 2 | 1.05 (0.34; 1.34) | 0.673 | 1.05 (0.82; 1.34) | 0.711 |  |
| Quintile 3 | 1.05 (0.83, 1.33) | 0.682 | 1.05 (0.83, 1.31) | 0.706 |  |
| Quintile 4 | 1.08 (0.84, 1.37) | 0.559 | 1.06 (0.82, 1.40) | 0.622 |  |
| Quintile 5 (highest sugar) | 1.04 (0.80, 1.34) | 0.771 | 1.07 (0.38, 1.29) | 0.616 |  |
| Linear effect | 1.00 (0.98, 1.03) | 0.793 | 1.00 (0.98, 1.03) | 0.690 | |
|  |  |  |  |  |  |
|  |  |  |  |  |  |

1. Data adjusted for ethnicity, maternal level of education, parental social class and maternal AUDIT score [↑](#footnote-ref-1)
2. No interaction was identified for the AUDIT variable and gender [↑](#footnote-ref-2)
